# UAV-Assisted Dynamic Clustering of Wireless Sensor Networks for Crop Health Monitoring

**DOI:** 10.3390/s18020555

**Published:** 2018-02-11

**Authors:** Mohammad Ammad Uddin, Ali Mansour, Denis Le Jeune, Mohammad Ayaz, el-Hadi M. Aggoune

**Affiliations:** 1 Lab STICC, ENSTA Bretagne, Brest 29200, France; ali.mansour@ensta-bretagne.fr (A.M.); denis.le_jeune@ensta-bretagne.fr (D.L.J.); 2 Sensor Networks and Cellular Systems Research Center, University of Tabuk, Tabuk 71491, Saudi Arabia; ayazsharif@ut.edu.sa (M.A.); hadi.aggoune@gmail.com (e.-H.M.A.)

**Keywords:** dynamic clustering, cluster head selection, IoT for agriculture, UAVs for agriculture

## Abstract

In this study, a crop health monitoring system is developed by using state of the art technologies including wireless sensors and Unmanned Aerial Vehicles (UAVs). Conventionally data is collected from sensor nodes either by fixed base stations or mobile sinks. Mobile sinks are considered a better choice nowadays due to their improved network coverage and energy utilization. Usually, the mobile sink is used in two ways: either it goes for random walk to find the scattered nodes and collect data, or follows a pre-defined path established by the ground network/clusters. Neither of these options is suitable in our scenario due to the factors like dynamic data collection, the strict targeted area required to be scanned, unavailability of a large number of nodes, dynamic path of the UAV, and most importantly, none of these are known in advance. The contribution of this paper is the formation of dynamic runtime clusters of field sensors by considering the above mentioned factors. Furthermore a mechanism (Bayesian classifier) is defined to select best node as cluster head. The proposed system is validated through simulation results, lab and infield experiments using concept devices. The obtained results are encouraging, especially in terms of deployment time, energy, efficiency, throughput and ease of use.

## 1. Introduction

The use of Internet of Things (IoT) technology to collect up-to-date information from crop fields to protect it from any kind of damage is the main objective of this research. Dynamic clustering is used to collect versatile data under harsh conditions. Clustering is an important way of increasing network lifetime and reliability. Many clustering techniques are proposed, which can be classified into four broad categories: static-sink static-nodes (Low-energy Adaptive Clustering Hierarchy (LEACH) and Hybrid Energy-Efficient Distributed clustering (HEED) [1,2]), mobile-sink static-nodes (Rendez-vous base routing [3,4,5,6]), static-sink mobile-nodes (cellular network [7]), and mobile-sink mobile-nodes (ad-hoc routing [8]). This research focuses on the mobile sink static nodes clustering method as all agriculture sensors are assumed to be static and we consider a mobile UAV to collect data from crop fields. For static sensor nodes, researchers propose predefined clusters and cluster head schemes to collect data. This type of clustering is not feasible in our case because of the frequent fluctuations of the sensor nodes. The situation becomes more critical, if the cluster head (CH) is down and the whole network becomes unfunctional. In addition, the path of the UAV is dynamic and sensor nodes are unaware of it; in this case, it rarely happens that a predefined CH lays on the path of the UAV and has a good link to it. Network-defined and Rendez-vous base clustering are also proposed in the literature, where all the nodes send periodic updates to maintain up-to-date CH locations or Rendez-vous Points (RVPs) from where an UAV can collect data. In this situation, the main drawback is the overhead for all nodes to update the CH locations continuously which results in battery drainage and reduces the network lifetime. Besides, UAVs have to search for and track the network-assigned CHs (Rendez-vous), which will affect the throughput of the system and deflect the UAV from its path. To the best of our knowledge, none of previously published clustering schemes considers the UAV path as a clustering criterion.

We proposs a dynamic clustering scheme. All the field sensor nodes are initially considered indistinguishable (no potential CH), so the the UAV sends a beacon message to activate all nodes residing in its vicinity, forming a cluster by considering its path and type of required data. The next step is to choose one node as the CH, merge all the cluster data on this point, and locate and connect it with the UAV at some reasonable height and distance.

IoT for agriculture is proposed in many research articles like [9,10,11], where they elaborate how sensors are useful in this field, what are the potential areas of application and how to collect and process information. To the best of our knowledge none of them provide in depth details like how to form clusters, select a CH, proper utilization of UAVs, independent movement of UAVs and energy preservation of the nodes. The most important aspect of our article is the evaluation of the proposed system through intensive simulations and real life experiments.

The rest of the paper is organized as follows: Section 2 provides a short background of the developed system. Section 3 and Section 4 present our dynamic cluster formation and cluster head selection techniques. The proposed three layer architecture is described in Section 5, while the system algorithm is given in Section 6. Different system parameters and their characteristics are defined in Section 7. The developed system in evaluated by using simulation models and IoT devices in Section 8 and Section 9, respectively. Finally Section 10 concludes this article.

## 2. Background

Data collection from Wireless Sensor Networks (WSNs) installed in farm fields faces many challenges like extreme climatic conditions characterized by high temperatures, dry air, dust/sand storms, lack of infrastructure, remote and huge geographical locations, etc. We propose a dynamic data collection mechanism to overcome the above mentioned challenges. In this study, smartness in agriculture is achieved in four steps:

1. Deployment of heterogeneous sensor nodes: a large number and wide range of heterogeneous sensors are used to monitor different parameters related to crop, soil and environment.
2. Use of Unmanned Aerial Vehicles (UAVs): UAVs are utilized to build the communication infrastructure between sensing devices and end-users.
3. Dynamic clustering: Clustering is the most important aspect of our study. It is the process used to arrange different sensing devices in groups according to geographical area, required data, path of the UAV, communication limitations, similarities or any other criterion.
4. Dynamic cluster head selection: Once available alive sensors have arranged themselves in a cluster formation then the next challenge is how to select a node as CH which will collect all the data from neighboring nodes and transmit it to the UAV. Selection of the CH is a tricky task, as the node having the best specifications and more suitable for the UAV (i.e., near to the UAV path) has to be selected as CH.

The developed system has many advantages over conventional ones. First of all it is a quickly deployed handy system which can be employed anywhere without any existing infrastructure. A farmer can collect needed data from an area of interest by using this system as all the data from the whole area is rarely needed. The mission is established for the UAV in advance in the form of waypoints to collect data from selective sensors and area. Network life can be improved massively as we propose the use of dynamic clustering, hence there is no need to discover and maintain data routes continuously. In WSNs, the node energy is mostly exhausted in broadcasting periodic updates to keep the network alive all the time. In our system, the network is formed only when data really needs to be collected and only selected nodes will participate. The use of UAVs also helps us to prolong the network life, as it enables us to collect data by visiting the IoT nodes at a suitable pre-arranged height. We are introducing a dual frequency communication system to further reduce the energy initialization. A low frequency (e.g., 433 MHz) is used to locate, navigate and shake-hand with sensor nodes while the high bandwidth and more energy demanding transceiver (e.g., 2.4 GHz) is switched on only when data need to be transmitted. Sensor nodes don’t have GPS modules and their reduced working loads are two other factors helping to optimize the node energy. Another important benefit of our proposed system is that it can survive and work well under harsh conditions, as sensor nodes are often fluctuating due to bad weather, and becoming covered by sand, water, mud or plant follicles. Pre-defined CHs and routes are not feasible, and in dynamic clustering only available nodes participate and the best among them will act as CH and formulate a temporary network to deliver the required data to the UAV.

**Figure 1:**
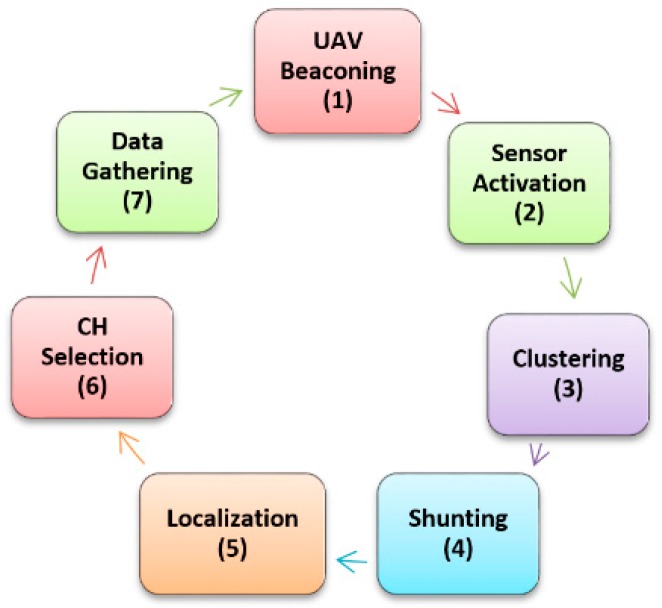
Proposed System lifecycle.

The life cycle of the proposed system is composed of seven steps, as shown in Figure 1. The UAV, which acts like data mule and the means of communication among the sensors, is the main part. The system life cycle starts when the UAV initiates the process of data gathering by sending a beacon message in step-1. Type of nodes, suggested data collection height and threshold to limit number of UAV-node connections are mentioned in this beacon. The nodes addressed in the UAV beacon are activated in step-2. In step-3 clusters are formed in the path of the UAV to preserve its predefined path. There is a possibility that none of the desired sensors have the capability to communicate with the UAV because of limited resources, while on the contrary, the UAV may get many responses from activated ground sensors and be unable to handle them at the same time. To tackle both of these conditions, step-4 is introduced to select some reasonable amount of field sensors for further processing. We name “shunting” this process of pushing or pulling some nodes from the process to make sure they are in a reasonable range. Localization (step-5) is to find out sensor nodes installed in crop field with the UAV, and a special lightweight energy-efficient antenna is designed for this purpose. We have given full details about this virtual phase array antenna in a previous article [12]. The best node among all will be selected as CH in step-6. Many parameters like energy, antenna size, energy consumption rate and distance to the UAV are investigated before selecting a node as CH. The final step-7 is data collection, in which the CH collects data from all neighboring nodes and the aggregated data is transmitted to the UAV by using a point to point dedicated link. This lifecycle keeps on until the whole or the selected area of the crop field is scanned and data is harvested successfully. The whole process spans three dimensions: dynamic clustering, dynamic cluster head selection and localization of field sensors by the UAV.

## 3. Proposed UAV Assisted Dynamic Clustering Architecture

As described above, the developed dynamic clustering consists of two components: sensor nodes and UAVs. No special CHs are installed/defined and all sensor nodes are considered indistinguishable.

### 3.1. UAV

In the developed system, the UAV plays a vital role in cluster formation, localization, communication and data gathering and the whole system is trigged by the UAV beacon. The UAV under consideration is a quad copter equipped with a localization system with the following specifications:

- Minimum operating flight height = 20 m,
- Maximum operating flight height = 500 m,
- Maximum speed = 7 m/s.

**Figure 2:**
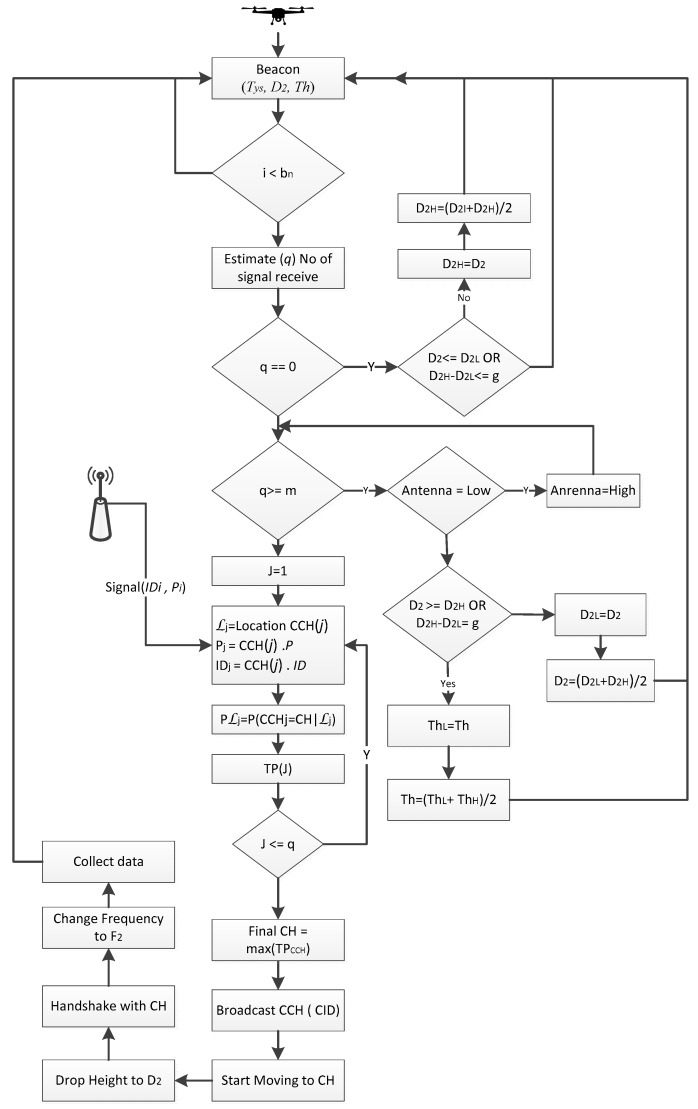
Flow chart of the UAV working.

The role of UAV is very critical in the developed system hence it is designed in such way that it bears most of the processing burden to simplify the tasks of the sensor nodes. It performs many key functions throughout its working cycle: activating the sensor nodes, shunting them for further processing, getting nodes’ initial information, locating all activated nodes, evaluating them, nominating the CH and finally collecting data from the selected CH. The working cycle of the UAV is given in Figure 2. 

The UAV sends a beacon message (see Table 1) to activate sensor nodes, and the parameters set in the beacon are as follows:

- *Sensor type*: Type of sensor nodes or combination need to be activated in response of this beacon.
- *Height*: it is current data collection height of UAV not the flying height. The sensor nodes that are capable to communicate at this distance will be considered as candidate for a CH.
- *Threshold*: This threshold will be used to limit the number of sensor nodes contesting for CH.
- *Trailer*: A trailer contains Error Detection Code (EDC) or any other information.

### 3.2. Sensor Node

Field sensors installed in the field to monitor a specific parameters about crop, soil or the environment have the following properties:

- Location-unaware, cheap in cost and left unattended.
- Support for multiple frequencies (at least two frequencies—433 MHz and 2.4 GHz—for localization and data transmission.
- By default, 433 MHz is switched on to hear the beacon from the UAV, afterward it will be used for localization and synchronization, while the 2.4 GHz transceiver will be activated on demand for communication only.
- Can hear the UAV if in range, but all might not be able to connect with it because of their internal parameters.
- Maximum communication range = 500 m.
- Processing and memory enabled.

All sensor nodes are considered to have a unique ID of the format given below:

- *Type*: We consider three basic types: crop, soil and environment, but we allocate 1 byte for further and future extensions.
- *Subtype*: Leaf, stem, root and any combination.
- *Purpose*: Temperature, humidity, thickness, flow and all possible combinations.
- *Unique ID*: Unique number of each sensor node.

The field sensor nodes will have 6 bytes of unique ID as shown in Table 2. The UAV will select any particular type or combination with the help of this ID, also called prefix. Sensor nodes will also use it to send beacon acknowledgement/reply messages.

In our proposed system, initially all sensor nodes are considered as undistinguishable (no special CH). A sensor node has to maintain five parameter values about its health:

1. *Energy*: How much energy is remaining,
2. *Consumption rate*: The consumption rate of energy to perform its job,
3. *Renewable energy*: Whether renewable energy is available or not,
4. *Antenna size:* Antenna size to estimate its communication range,
5. *Data size*: How much data should be transmitted.

**Figure 3:**
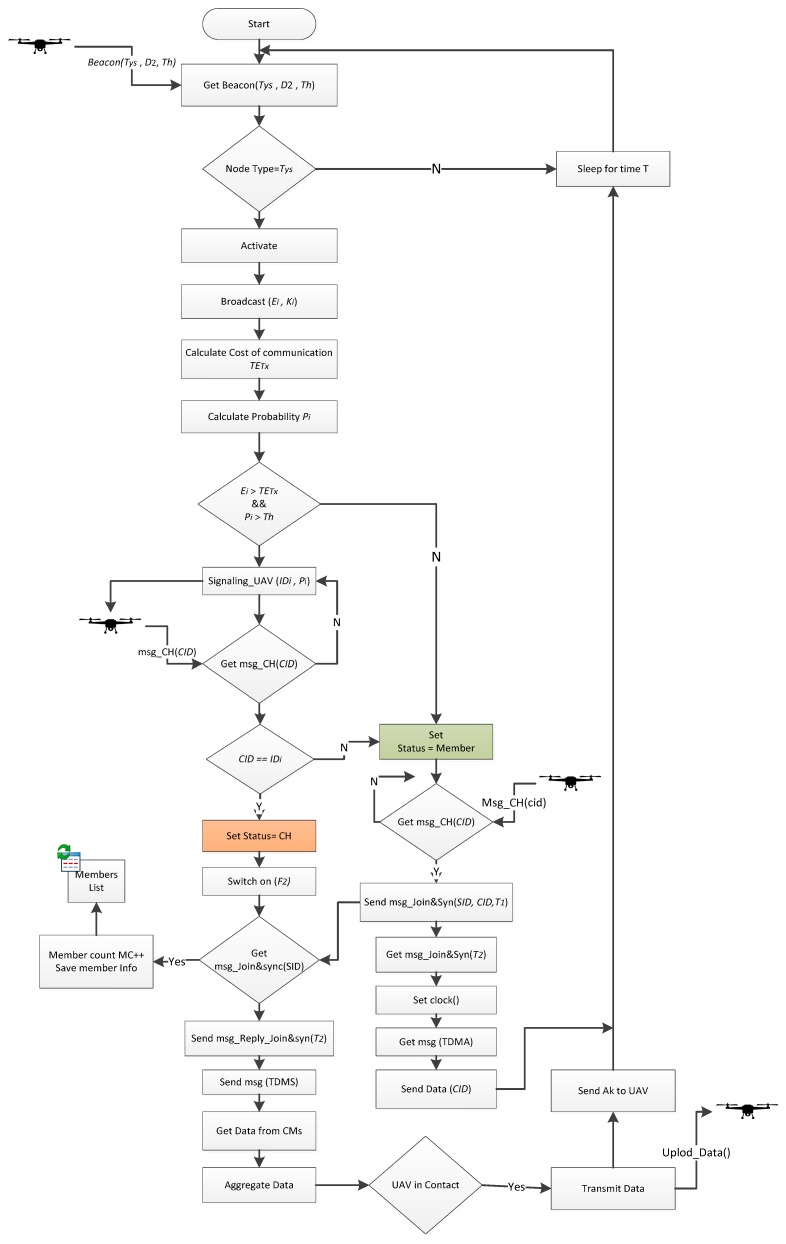
Working flow of sensor node.

All activated sensor nodes will calculate a probability value to become a CH based on these health indicators. The sensor nodes’ reply to the UAV will consist of 9 Bytes containing a prefix (node ID) in the packet header, a probability value (the probability to be selected as a CH, calculated by the Bayesian formula by putting its entire health indicator’s values) in payload and trailer. The 1 trailer byte is allocated for Cyclic Redundancy Check (CRC) and future use. A sensor node activated in response to an UAV beacon will send a reply shown in Table 3: 

The most important component of our developed system is the sensor node. The whole system is developed in such a way to facilitate the tasks of this component and let it optimize its resources to monitor the crop parameters for a longer time. In our developed system, the daily routine working of sensor node is limited to sense and sleep. It becomes only active for communication after receiving a beacon message from the UAV. The sensor node working process is expressed in an algorithmic form as shown in Figure 3. The process starts when the node receives a beacon then it calculates its Bayesian probability to be a CH and informs the UAV about it. The UAV then selects one of the activated nodes as CH, which should collect data from the whole cluster, aggregate and forward it to the UAV. The list of variables used in the rest of this article is given in Table 4.

## 4. Dynamic Cluster Head Selection

Once the UAV assisted cluster is formed, the developed system will grade cluster nodes into two types: the first type contains the nodes that don’t have the capability to approach the UAV, called cluster members (CMs); candidate cluster heads (CCHs) are the other type. The CCHs are further shunted by the developed system to keep them in a range from *1* to *N* (where *N* is the maximum capacity of the UAV to locate sensor nodes). All CCHs and the UAV will collectively take part in the selection process to nominate a node as CH. 

**Figure 4:**
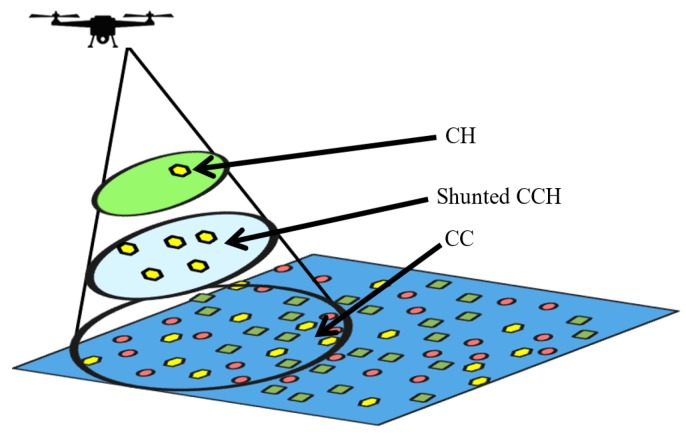
Dynamic clustering scheme.

Many parameters (like remaining energy, available renewable energy, energy consumption rate, antenna size and distance to the UAV) are considered in this selection process. In the proposed system, the tasks of cluster formation and the CH selection are conducted dynamically at runtime according to the context and then a reliable point to point backbone connection is established between the CH and the UAV to collect all required data for further processing and decision making. The proposed dynamic clustering scheme is illustrated in Figure 4.

Each node will participate in the CH selection process depending upon its probability calculated by using a Bayesian classifier. Bayesian probability has been studied for two centuries and many researchers are using it for different purposes [13,14,15,16]. We are inspired by Bayesian spam filtering [17] as it is more close to our problem domain. We derived our probabilities in as follows:

Let us suppose there are n sensor nodes $S=\left(s_{1},\;s_{2},\ldots ,\;s_{n}\right)$ and each sensor node $s_{i}$ has z attributes (independent variables) represented by a vector $A=\left({𝒶}_{1},{𝒶}_{2},\ldots ,\;{𝒶}_{z}\right)$. A sensor node $s_{i}$ can be in one of two states:

(a) Cluster Head (CH)
(b) Cluster Member (CM)

represented by State=(CH,CM)

$P\left(s_{i}=\mathrm{CH}|{𝒶}_{ij}\right)$ is the posterior probability of a given sensor node $s_{i}$ to be a CH knowing its attribute ${𝒶}_{ij}$.

$P\left(s_{i}=\mathrm{CH}\right)$ is the prior probability of given node to be a CH.

$P({𝒶}_{ij}|s_{i}=\mathrm{CH})$ is the likelihood probability that the highest value of the attribute ${𝒶}_{ij}$ is in CH node.

$P\left(s_{i}=\mathrm{CM}\right)$ is the prior probability that a given node $s_{i}$ is a member node.

$P({𝒶}_{ij}|s_{i}=\mathrm{CM})$ is the probability that the highest value of attribute ${𝒶}_{ij}$ is in CM and not in CH.

$P\left({𝒶}_{ij}\right)$ is the prior probability of the attribute value ${𝒶}_{ij}$ is the highest one.

Equation (1) is the probability of the node $s_{i}$ to be a cluster head by knowing only one parameter ${𝒶}_{ij}$. If all parameters $A_{i}=\left({𝒶}_{1},{𝒶}_{2},\ldots ,\;{𝒶}_{z}\right)$ of node $s_{i}$ are independent in nature then the conditional probability of this node, by considering whole set $A_{i}$ can be calculated as:

(1) $$P\left({𝒶}_{i1},{𝒶}_{i2},\ldots ,{𝒶}_{iz}{|s}_{i}=\mathrm{CH}\right)=\overset{z}{\underset{j=1}{\prod }}P\left({𝒶}_{ij}{|s}_{i}=\mathrm{CH}\right)=\overset{z}{\underset{j=1}{\prod }}\frac{P\left(s_{i}=\mathrm{CH}|{𝒶}_{ij}\right)\;P\left({𝒶}_{ij}\right)}{P\left(s_{i}=\mathrm{CH}\right)}\;=\;\frac{\prod_{j=1}^{z}P_{ij}\;P\left({𝒶}_{ij}\right)}{{\left[P\left(s_{i}=\mathrm{CH}\right)\right]}^{z}}$$

(2) $$P\left({𝒶}_{i1},{𝒶}_{i2},\ldots ,{𝒶}_{iz}{|s}_{i}=\mathrm{CM}\right)=\overset{z}{\underset{j=1}{\prod }}P\left({𝒶}_{ij}{|s}_{i}=\mathrm{CM}\right)=\overset{z}{\underset{j=1}{\prod }}\frac{P\left(s_{i}=\mathrm{CM}|{𝒶}_{ij}\right)\;P\left({𝒶}_{ij}\right)}{P\left(s_{i}=\mathrm{CM}\right)}=\frac{\prod_{j=1}^{z}\left(1-P_{ij}\right)\;P\left({𝒶}_{ij}\right)}{{\left[1-P\left(s_{i}=\mathrm{CH}\right)\right]}^{z}}$$

If:

$P_{i}$ is the probability $P\left(s_{i}=\mathrm{CH}|A_{i}\right)$ of the *i*th node, $s_{i}$ to be a CH by knowing a set of all its parameters $A_{i}$, $P_{ij}$ is the probability $P\left(s_{i}=\mathrm{CH}|{𝒶}_{ij}\right)$ of the *i*th node, $s_{i}$ to be a CH by knowing its *j*th parameter ${𝒶}_{ij}$ from the parameters set $A_{i}$ and ${𝒶}_{ij}$ is the *j*th attribute of the *i*th sensor node.

Then:

(3) $$P\left(s_{i}=\mathrm{CH}|A_{i}\right)=\frac{P\left(s_{i}=\mathrm{CH}\right)\;P\left(Ai{|s}_{i}=\mathrm{CH}\right)\;}{P\left(A_{i}\right)}=\frac{P\left(s_{i}=\mathrm{CH}\right)\;P\left(A_{i}{|s}_{i}=\mathrm{CH}\right)\;}{P\left(s_{i}=\mathrm{CH}\right)\;P\left(A_{i}{|s}_{i}=\mathrm{CH}\right)\;+P\left(s_{i}=\mathrm{CM}\right)\;P\left(A_{i}{|s}_{i}=\mathrm{CM}\right)}$$

(4) $$P\left(s_{i}=\mathrm{CH}|{𝒶}_{ij}\right)=\;\frac{P\left(s_{i}=\;\mathrm{CH}\right)\;P\left({𝒶}_{ij}{|s}_{i}=\mathrm{CH}\right)}{P\left({𝒶}_{ij}\right)}$$

(5) $$P\left(s_{i}=\mathrm{CH}|{𝒶}_{ij}\right)=\;\frac{P\left(s_{i}=\mathrm{CH}\right)\;P\left({𝒶}_{ij}{|s}_{i}=\mathrm{CH}\right)}{P\left(s_{i}=\mathrm{CH}\right)\;P\left({𝒶}_{ij}{|s}_{i}=\mathrm{CH}\right)\;+\;P\left(s_{i}=\mathrm{CM}\right)\;P\left({𝒶}_{ij}{|s}_{i}=\mathrm{CM}\right)}$$

Putting the values of (1) and (2) in Equation (5):

$$P\left(s_{i}=\mathrm{CH}|A_{i}\right)=\;\frac{\;P\left(s_{i}=\mathrm{CH}\right)\;\frac{\prod_{j=1}^{z}P_{ij}\;P\left({𝒶}_{ij}\right)}{{\left[P\left(s_{i}=\mathrm{CH}\right)\right]}^{z}}\;}{\;P\left(s_{i}=\mathrm{CH}\right)\frac{\prod_{j=1}^{z}P_{ij}\;P\left({𝒶}_{ij}\right)}{{\left[P\left(s_{i}=\mathrm{CH}\right)\right]}^{z}}+\left[1-P\left(s_{i}=\mathrm{CH}\right)\right]\frac{\prod_{j=1}^{z}(1-P_{ij})\;P\left({𝒶}_{ij}\right)}{{\left[1-P\left(s_{i}=\mathrm{CH}\right)\right]}^{z}}\;}$$

$$P\left(s_{i}=\mathrm{CH}|A_{i}\right)=\;\frac{\frac{1}{P{\left(s_{i}=\mathrm{CH}\right)}^{z-1}}\;\;\prod_{j=1}^{z}\;P_{ij}\;P\left({𝒶}_{ij}\right)\;}{\frac{1}{P{\left(s_{i}=\mathrm{CH}\right)}^{z-1}}\;\;\prod_{j=1}^{z}\;P_{ij}\;P\left({𝒶}_{ij}\right)\;+\;\frac{1}{{\left[1-P\left(s_{i}=\mathrm{CH}\right)\right]}^{z-1}}\prod_{j=1}^{z}\;(1-P_{ij})\;P\left({𝒶}_{ij}\right)}$$

Let us consider that $P\left({𝒶}_{ij}\right)$ are constant then the previous equation can be simplified as follows:

(6) $$P\left(s_{i}=\mathrm{CH}|A_{i}\right)=\;\frac{\prod_{j=1}^{z}\;P_{ij}\;\;}{\;\prod_{j=1}^{z}\;P_{ij}\;+\;{\left(\frac{P\left(s_{i}=\mathrm{CH}\right)}{1-P\left(s_{i}=\mathrm{CH}\right)}\right)}^{z-1}\prod_{j=1}^{z}\;(1-P_{ij})}$$

If considering “not biased” condition where all nodes in the network have the same probability to become cluster head then Equation (6) can be written as:

(7) $$P\left(s_{i}=\mathrm{CH}|{𝒶}_{ij}\right)=\;\frac{P\left({𝒶}_{ij}{|s}_{i}=\mathrm{CH}\right)}{P\left({𝒶}_{ij}{|s}_{i}=\mathrm{CH}\right)+P\left({𝒶}_{ij}{|s}_{i}=\mathrm{CM}\right)}$$

and Equation (7) can be written as:

(8) $$P_{i}=P\left(s_{i}=\mathrm{CH}|A_{i}\right)=\;\frac{P_{i1}.P_{i2}\ldots \ldots P_{iz}}{P_{i1}.P_{i2}\ldots \ldots P_{iz}+\;(1-P_{i1}).\left(1-P_{i2}\right)\ldots \ldots (1-P_{iz})}$$

As Equation (8) may cause a floating point underflow problem, we convert the equation into the log domain:

(9) $$\begin{array}{c} \frac{1}{P_{i}}-1=\;\frac{\left(1-P_{i1}).\left(1-P_{i2}\right)\ldots \ldots (1-P_{iz}\right)}{P_{i1}.P_{i2}\ldots \ldots P_{iz}\;}\;\Rightarrow \\ \ln\left(\frac{1}{P_{i}}-1\right)=\;\overset{z}{\underset{j=1}{\sum }}\left(\ln(1-P_{ij})-\ln P_{ij}\right) \end{array}$$

$\ln\left(P_{ij}\right)$ can produce a problem when $P_{ij}$ is close to zero; the results will be asymptotically correct.

Let $\mu =\;\overset{z}{\underset{j=1}{\sum }}\ln(1-P_{ij})-\ln P_{ij}$:

$$\mu =\;\overset{z}{\underset{j=1}{\sum }}\ln\left(\frac{1-P_{ij}}{P_{ij}}\right)=\;\overset{z}{\underset{j=1}{\sum }}\ln\left(\frac{1}{P_{ij}}-1\right)$$

Finally, we can write that:

(10) $$P_{i}=\;\frac{1}{e^{\;\mu }+1}$$

## 5. Three-Layer Architecture of the Developed System

**Figure 5:**
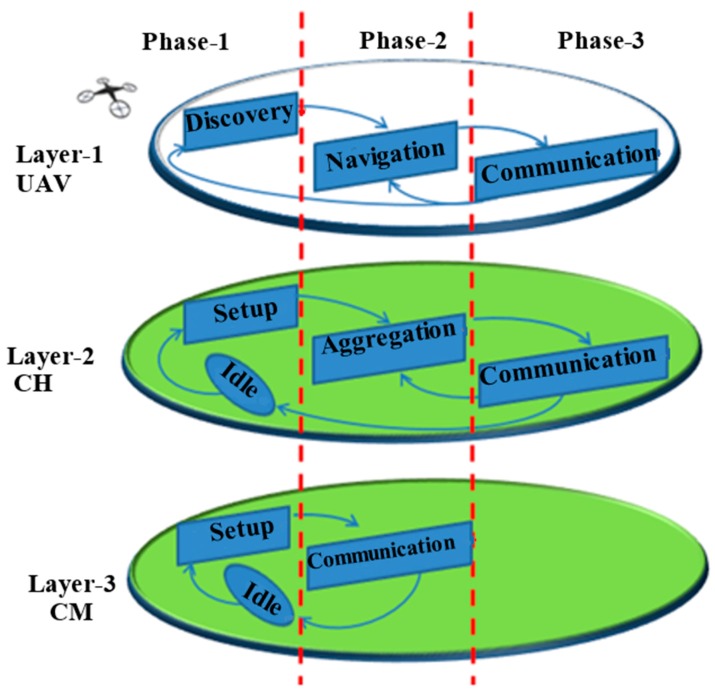
Multi-layer and multi-phase proposed system architecture.

The proposed UAV assisted routing and data gathering scheme is developed in multi-layers and multi-phases (see Figure 5):

1. Layer-1 UAV: UAV is the main part and the top layer,
2. Layer-2 CH: One node per cluster will be selected as CH and it will form a 2nd middle layer,
3. Layer-3 CM: Composed of ground sensor nodes.

Each layer further is divided into three phases as shown in Figure 5. The main part of this system is the UAV acting like a data mule, and the process is started by its beacon message. The UAV can operate at two different frequencies: very low power and long-range UHF $F_{1}$ used for localization and high data rate WiFi $F_{2}$ for communication. The UAV is performing localization at distance $D_{1}$ which is considered as constant and should be high enough to satisfy the far field conditions of $F_{1}$. Data collection is conducted at distance $D_{2}$ which can be adjusted from 20 to 170 m by considering the CH transmission ability, safe flight distance and WiFi limitations. Top layer (UAV) layer is composed of three phases discuded in detail in next subsection.

### 5.1. Discovery

**Figure 6:**
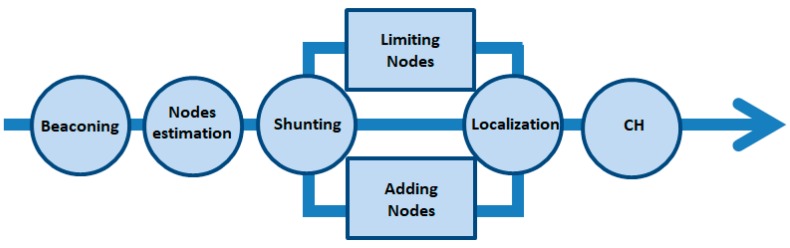
UAV discovery phase.

The UAV performs five main tasks in this phase including: beaconing, estimating the number of connected nodes, shunting, localizing and CH nomination, as explained in Figure 6.

Let us suppose that the UAV is flying at a height $D_{1}$ and initiates its data gathering process by sending a beacon $B\left(T_{ys},D_{2},Th\right)$ containing the type $T_{ys}$ of nodes required to activate for data collection, distance $D_{2}$ at which it will collect data and Th which is the threshold to limit the activated nodes who will contest for the role of CH. Initially Th=0 means all nodes can participate in the CH selection process. Suppose that there are n sensor nodes $S=\left\{s_{1},\;s_{2},\ldots ,s_{n}\right\}$ installed in a crop field. Assume O nodes become activated in response of UAV beacon such that 1≤O≤n and $SS=\left\{SS\subseteq S|SS\in T_{ys}\right\}=\left\{ss_{1},\;ss_{2},\ldots ,\;ss_{O}\right\}$. SS is the set of nodes that will make cluster in response of this beacon.

If each active node $SS_{i}$ has ${𝒷}_{i}$ bit data to transmit then T𝒷 represents the total size of cluster data, that is expected to be transmitted by the CH to the UAV:

(11) $$T𝒷=T𝒷\left(SS\right)=\sum_{i=0}^{O}{𝒷}_{i}$$

The energy required to transmit T𝒷 to UAV for distance *D*_2_ is:

(12) $$TE_{Tx}=\left(E_{elec}\times T𝒷\right)+\left(E_{amp}\times T𝒷\times {\left(D_{2}\right)}^{2}\right)$$

where $E_{elec}$ is the energy being dissipated to run the transmitter and $E_{amp}$ is the energy dissipation to amplify the message up to D m.

While UAV is in the discovery phase, activated ground sensors SS will be in setup phase to form a cluster. Every member of SS will calculate a Bayesian probability $P_{i}$ considering all its parameters (energy, consumption, antenna size, data size, etc.). A detailed discussion on Bayesian probability is given in Section 4. Based on $E_{i}$, the energy of the node $SS_{i}$, and $P_{i}$, the probability to be selected as CH, a further subset called candidate cluster heads of CCH. $CCH=\left\{cch_{1},\;cch_{2},\ldots ,cch_{q}\right\}$, where 1≤q ≤O, is formed such that:

(13) $$CCH\;=\left\{CCH\;\subseteq SS\;|\forall \;E_{i_{current}}\;T𝒷\;\&\;P_{i}\rangle Th\right\}$$

where $E_{i_{current}}$ is the current energy of node i.

As described earlier, Th=0 means any activated node having enough energy can be a member of the CCH.

All CCH nodes start sending beacon replies to the UAV in the form of narrowband signals. At this point, UAV estimates the number of CCH (|CCH|=q). If the UAV estimates that q=0 or q≥m−1, where m is the number of antenna elements on board, then the shunting process is started to keep the candidate cluster heads within a reasonable range; Otherwise, the UAV will locate all CCH nodes by using a special virtual phase array antenna developed for said purpose (a detailed model is given in Section 4). Based on CCH location and $P_{i}$ values, the UAV nominates a final CH and broadcasts a message to all nodes of set SS to inform them about the CH nomination.

Shunting is the important process of discovery phase to handle the situation when:

q=0 or q≥m

If q=0 or CCH=∅, it means no CCH member has the capability to send aggregated data to the UAV; In this case, shunting decreases $D_{2}$ in steps down to a minimum height depending upon safe flight constraints.

If q≥m means there are many good CCH nodes and UAV cannot locate all at once; in this case, shunting can take three steps:

- Increase antenna capacity from m to 2m,
- Increase $D_{2}$ in steps up to the limit of $F_{2}$,
- Increase Th.

Once a cluster head is selected the next phase of the UAV is navigation.

### 5.2. Navigation

Once a CH is selected the UAV enters the next phase called navigation. In similar way, the CH and CM switch their phases from setup to aggregation and communication, respectively. In this phase, all active nodes will switch on their frequencies from $F_{1}$ to $F_{2}$ (433 MHz to 2.4 GHz). 

**Figure 7:**
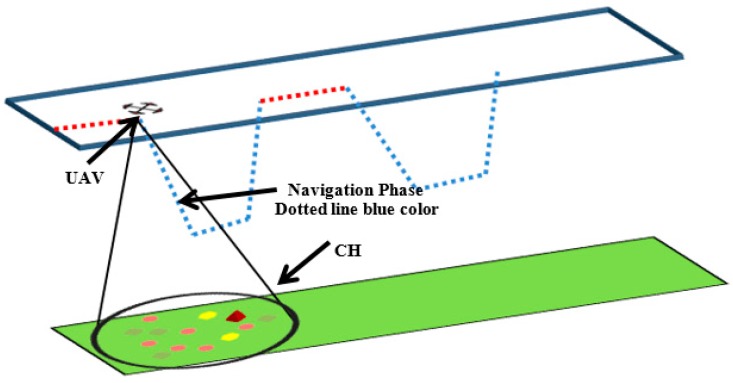
Navigation phase of the proposed system.

Only the CH will operate on both frequencies. CH will use *F*_2_ to collect data from CMs and $F_{1}$ for UAV navigation. While the UAV approaches the CH and attains an agreed height $\;D_{2}$ all CM nodes must transmit their data to the CH which will aggregate it and get ready to make a link with the UAV. As soon as the UAV approaches $D_{2}$ and starts handshaking, the CH will switch off its $F_{2}$ module and the navigation phase is over. The navigation diagram is elaborated in the diagram of Figure 7.

### 5.3. Communication

**Figure 8:**
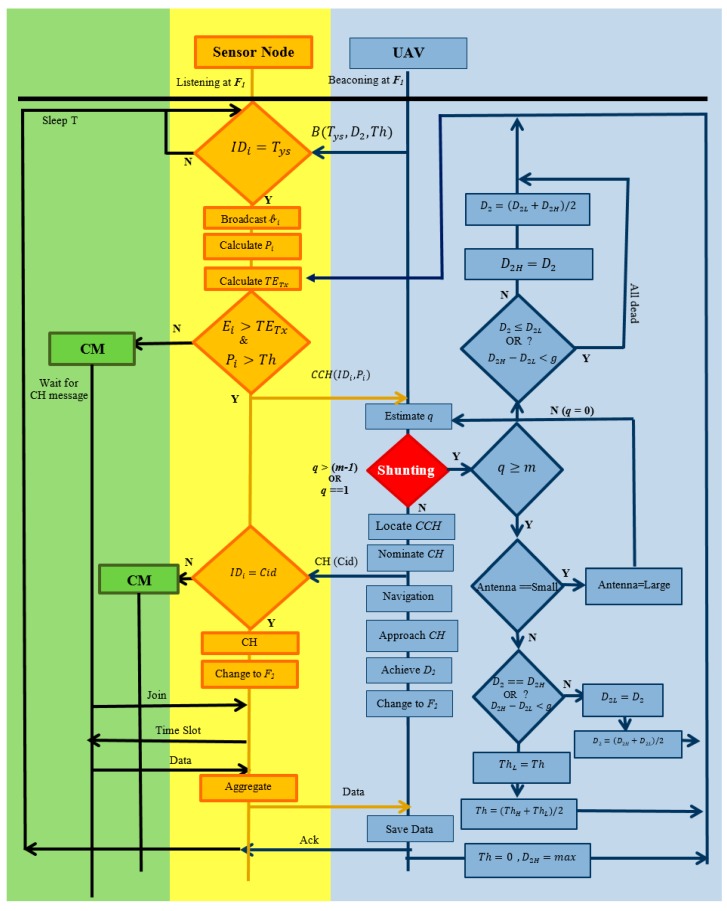
State diagram of the developed system.

In the last phase, only the top two layers (UAV and CH) of our developed system will participate. The CH will transmit the whole data to the UAV at the frequency $F_{2}$; once transmission is done, the CH will go to sleep for a specific time T, while all CMs had already been shifted to sleep mode. To elaborate the exact sequence of steps the flow chart diagram of the developed system is shown in Figure 8.

## 6. Developed System Algorithm

The overall working of the developed system is explained in the state diagram (Figure 8) where the homological sequences of steps are shown. The variables used in the algorithm are listed in Table 4. As shown in Figure 8, blue color represents the top layer, Yellow is the middle one and green represents the last layer.. The UAV needs the following inputs to start the mission and data collection procedure: the path of the UAV consists of waypoints {(x1,y1), (x2, y2), (x3, y3)….}, $D_{1}$ height, the UAV normally flies at this height and conducts localization and navigation (e.g., 200), $D_{2h}$ is maximum possible height for data collection (e.g., 170 m), $D_{2L}$ is the lowest possible height for the data collection (e.g., 20 m), $D_{2}$ is the data collection height, and the UAV only obtains this height to establish a backbone high data rate link with the CH to collect data, after that UAV goes back to its normal height $D_{1}$.

Initially $D_{2}$ should mainly be set to an average height (e.g., 170 + 20/2 = 95 m), Th the threshold value 0 ≤Th≤1, $Th_{L}$ denotes minimum threshold, $Th_{H}$ stands for maximum threshold and g is the GPS accuracy (e.g., 5 m).

The UAV is equipped with a virtual phase array antenna which can operate at two different modes Low/High, where Low means with the least specification that can locate fewer targets and High means maximum specifications [12].

The UAV sends beacon messages to activate the ground sensors. The beacon is composed of three basic kinds of information: $T_{ys}$ is the type of nodes must be activated, $D_{2}$ distance at which UAV will collect data and Th is threshold. The process steps are as below:

1. The nodes not mentioned in this beacon message continue sleeping, while others will become activated.
2. The activated nodes will broadcast a message to all their neighbors to let them know how much data and energy they have.
3. All activated nodes will calculate the cost of transmission $TE_{Tx}$ as per Equation (12) and their probability to be a cluster head $P_{i}$ as per Equation (10). All the nodes having energy greater than $TE_{Tx}$ and get a higher probability than the threshold will declare themselves candidate cluster heads (CCHs) and remaining ones will be cluster members (CM).
4. CCHs will proceed further and start sending a narrowband signal to UAV having $P_{i}$ and their IDs.
5. When the UAV gets a reply from ground sensor nodes, it will use its on board virtual antenna and estimate the number of replying sensor nodes q. If the UAV finds 0 < q < m then it will go into localization mode, to estimate the location of these CCHs, otherwise it will start the shunting process.
6. Shunting is a process to pull or push some sensor nodes from the process to keep their number within some reasonable range (1  to  m−1)*,* m is the total number of virtual antenna elements. The shunting process is shown by a red diamond in the state diagram (see Figure 6 For shunting, UAVs have three options that it will be used in steps:
  - The first option: the UAV is equipped with an adjustable virtual antenna that can operate in two different modes: fast mode with minimum localization ability and high specification mode. If the UAV finds many CCH nodes contesting for cluster head (CH), it will switch its mode to high performance mode.
  - If the UAV finds that the number of replying CCHs is even greater than the high performance antenna’s capacity then it will try to reduce the number of CCHS by increasing the data collection height $D_{2}$. The $D_{2}$ can also be used in reverse order, if no sensor node is contesting for CH, it means none has the ability to send aggregated data to the UAV at this distance and in this case, $D_{2}$ can be decreased up to a minimum level (safe flight).
  - $D_{2}$ is always kept in the middle of highest height $D_{2H}$ and the lowest $D_{2L}$ as $D_{2}=\left(D_{2H}+D_{2L}\right)/2$. We are shrinking and expanding low and high values as per the requirements. If many CCHs were found and we want to limit them by increasing the height the low value is shifted to the middle and the middle value is calculated again. The same treatment in reverse order is used if no CCH is found, and the data collection height is decreased by shifting the high value to the middle and the middle is calculated again.
7. This $D_{2}$ height tuning process continues till:
  - The number of CCHs falls in the range (1 to m − 1)
  - $D_{2}$ reaches the boundary condition extreme high or low
  - The distance between high and low becomes less than the GPS accuracy of let’s say 5 m.
8. The third option is the variation of the threshold Th; by increasing Th from 0 to 0.5 roughly half of the nodes will withdraw themselves from CH selection. The UAV can increase Th at every iteration.
9. The UAV will localize the activated candidate cluster heads and select the best one as CH on the basis of $P_{i}$ information received and the distance to the next waypoint. The node having highest $P_{i}$ value and closer to the waypoint will be selected as CH.
10. The UAV will send CH messages to all activated nodes.
11. The nodes receiving a CH message will send a join request. The CH receives the join request and allocates a time slot.
12. The nodes receive the time slot and send its data.
13. The CH aggregates the data and transmits it to the UAV.
14. The UAV moves to next cluster. All active nodes finish their transmission, and go to sleep for a fixed time.

## 7. Characteristics of the Developed System

The characteristics of the proposed system including link budget and communication range are calculates to evaluate the system and to build a simulation model based on these real values.

### 7.1. Link Budget

The critical parameter in our application is the energy, in particular the energy required for the cluster heads to transmit their beacon and useful information to the UAV. The relationship among energy, distance and frequency is given in Equation (14) [18]:

(14) $$Path\;loss=32.45\;dB+20\;Log_{10\;}\left(frequency\;in\;MHz\right)+20\;Log_{10\;}\left(distance\;in\;Km\right)$$

We realize that at a large distance from the UAV, it is preferable to use a lower frequency to minimize the power consumption of the cluster head.

In our developed system, the UAV and sensor nodes are performing two major types of communication: one is a long range communication for identification/localization/navigation and other is a short range for the data exchange. For the long range communication, we don’t need a high bandwidth. We are using a bit lower frequency that can penetrate to larger distances with very low energy. High frequency (the required one is some standard such as WiFi) is only used for data exchange, as the data size may be large enough so we need high bandwidth. Short-range communication is more energy demanding and increases exponentially with increasing distance. In our developed system, we care about this transmitter receive distance. Link budgets for both the frequencies are defined in the next section.

### 7.2. Long Range Communication (433 MHz UHF Frequency)

A low power transceiver operating at 433 MHz frequency is suggested for long range communication activities like node localization, synchronization and handshaking. For all these activities, we don’t need high bandwidth, as only a few bits of data are needed. Let us assume a small radiated power (100  μW) is considered for transmission:

(15) $$PW=100\;\mu W=10\;log_{10\;}\left(PW\;\mathrm{mW}\right)\mathrm{dBm}=10\;log_{10}\left(0.1\right)\mathrm{dBm}=-10\mathrm{dBm}\;$$

Masking factor (by objects like vegetation or other) for UHF long distance is considered very small MF= 5 dB.

If receiver sensitivity of a UAV is  ξ= −130 dBm  and a signal to noise ratio necessary to demodulate is *SNR* = 15 dB.

The maximal path loss constraint is calculated as:

$$M_{PL}=PW-\;\xi -SNR-MF=-10+130-15-5=100\;\mathrm{dB}$$

Based on Equation (14), we can calculate the maximum distance covered by UHF communication:

$$20\;log_{10}\left(D_{1}\right)=100-32.45-20\log\left(F_{1}\right)=100-32.45-52.7=14.85$$

$$D_{1}=10^{0.7425}≅5.527\;\mathrm{Km}$$

The maximum range between the UAV and the cluster head node is $D_{1}\;=\;5.527\;$ Km.

In our developed system, we don’t need to communicate over a long distance of 5 Km. If we are considering a UAV that is flying at a height of 300 m and it wants to activate nodes in 500 m area then only 0.825 μ*W* of power is required by the UAV to transmit the $F_{1}$ signal to sensor nodes to communicate.

### 7.3. Short Range Communication (2.4 GHz WiFi Frequency)

The high power and short range (e.g., WiFi) module is only activated only when data needs to be transmitted. In a similar way, we can find the maximum distance covered by a WiFi signal taking all parameters as considered for $F_{2}$ frequency, except for a higher masking factor that is 20.

Receiver sensitivity of a UAV is ξ= −130 dBm

A signal to noise ratio necessary to demodulate is SNR= 15 dB

The maximum path loss constraint is:

$$M_{PL}=PW-\xi -SNR-MF=85\;\mathrm{dB}$$

Based on Equation (15), we can calculate the maximum distance covered by WiFi communication:

$$20\;log_{10}\left(D_{2}\right)=85\;dB-32\;dB-20\;log_{10}\left(F_{2}\right)=\left(85-32.45-67.6\right)dB=-15.05\;dB$$

$$\begin{array}{c} Log_{10}\left(D_{2}\right)=-\frac{15.05}{20}=-0.7525\Rightarrow \\ D_{2}=10^{-0.7525}≅0.176\mathrm{Km} \end{array}$$

The maximum range between the UAV and the cluster head node for data collection is 0.177  Km. We will then consider a maximum distance in the (*x*, *y*) plane of the order of $xy_{UAV}\;=\;150\;m$.

## 8. Simulation

Simulations are conducted in OMNeT++ (Objective Modular Network Test bed in C++) and MatLab to evaluate the performance of the developed system.

### 8.1. Simulation Model

**Figure 9:**
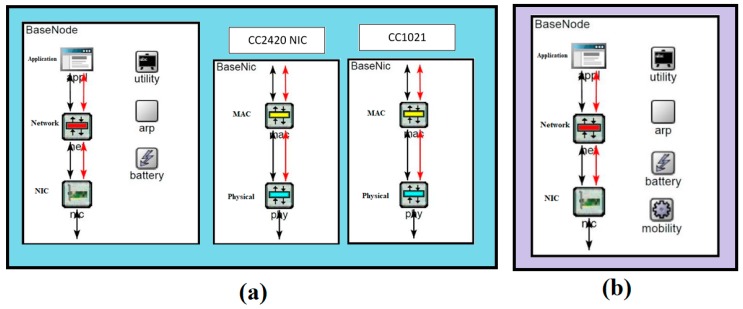
Developed OMNet++ simulation model (**a**) Sensor node simulation model (**b**) Mobile sink extra module other than sensor node model.

As per our designed protocol, a wireless sensor node is equipped with two different frequencies: 433 MHz for localization and identification of nodes and 2.4 GHz for data transmission. We developed a sensor node simulation model using two NICs CC2420 [19] and CC1021 [20] as shown in Figure 9a. Both NICs are composed of MAC and physical layer and the sensor node consists of three layers (Application, Network and NIC). CC2420 is a 802.15.4 compliant Network Interface Card (NIC) operating at 2.4 GHz frequency and having built-in CSMA/CA in MAC layer, while CC1021 is a low power RF transceiver for narrowband systems operating at 433 MHz. The sensor node is considering as smart enough that it can negotiate a communication height with the UAV by considering the amount of data that must be transmitted and the remaining energy power level. It utilizes only 0.825 μ*W* of energy while operating with the CC1021 for localization. The largest energy depletion factor is data transmission between the CH and UAV using a higher frequency, e.g., WiFi. If the considered node is selected as CH then the energy consumption of the CC2420 is optimized by limiting the agreed CH-UAV height. The UAV receives data at a lower height suitable for the CH. The UAV model is created with same NICs as in the sensor nodes. The only difference is the addition of mobility components in the main module as shown in Figure 9b.

The UAV is simulated to fly with a constant speed of 20 m/h and a height of 200 m. Initially, it switches on its CC1021 module and starts sending the beacon message. Once the UAV identifies the location of the selected CH, it starts moving towards it and reduces its height up to an agreed level. When the UAV approaches the CH, it will change its communication module from CC1021 to CC2420. All these simulation are available on YouTube links [21,22,23,24,25].

### 8.2. Simulation Cases

As described earlier, our case scenario belongs to the category of a Mobile Sink Static Node (MSSN) network where a sink is traversing the network to collect data while all sensor nodes are kept constant. If we are taking the sink as a mobile node then there are three possibilities:

(1) Direct data collection,
(2) Cluster-based data collection with a controlled path,
(3) Cluster-based data collection with an independent UAV path.

In this scheme, the UAV can move freely and instruct the ground sensor nodes to form clusters and help them to select a cluster head. To evaluate this developed system, we studies three cases, one for each type:

1. Direct Data Collection (DDC) by UAV, as described in [26],
2. Network Assisted Data Collection (NADC), as given in [27]
3. Our developed UAV Routing Protocol (URP).

One hundred sensor nodes are deployed randomly with a uniform distribution in a 2 Km^2^ area.

### 8.3. Simulation Results

Different simulation models and cases are executed a number of times (at least five times) and the average results are considered for more accuracy. Different simulation cases are described one by one as given below:

#### 8.3.1. Number of Dead Nodes vs. Simulation Time

In this simulation run, we wanted to check how energy is utilized and how fast the sensor nodes’ energy becomes completely exhausted. We simulate each case (DDC, NADC, URP) separately with same parameters such as number of nodes, simulation time, initial node energy, node/UAV communication ranges, data collection height, etc.

**Figure 10:**
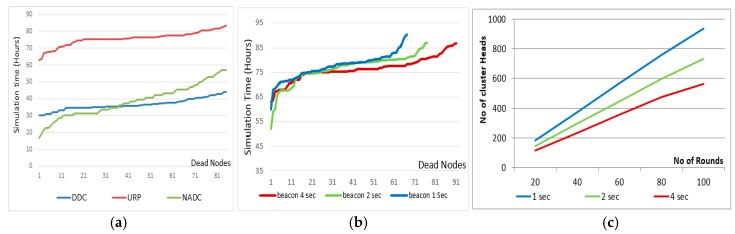
Simulation results of different cases (**a**) Number of dead nodes vs. simulation time (**b**) effect of varying beacon sending period.

We can conclude that using our developed system, the network lifetime can be improved by 20% or more on the expense of UAV energy, adding more intelligent sensor nodes and dual frequency support in the UAV and nodes (as Figure 10a).

#### 8.3.2. Effect of Beacon Sending Period

The UAV starts its data collection process by sending beacon messages to activate the sensor nodes. This beaconing period decides the size of clusters, and the longer the beaconing period the larger the cluster size. This phenomenon can also affect the performance of the system. The developed system is tested against varying beacon sending periods and the results are shown hereinafter.

**Figure 11:**
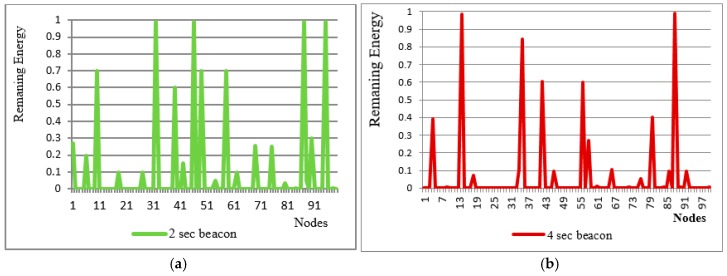
Effect of beaconing period on energy utilization (**a**) 2 s beaconing (**b**) 4 s becoming.

Figure 10b represents the total number of dead nodes on the X-axis, simulation time on the Y-axis and different curves show the performance of system using different beacon periods. The lower the curve the better is the performance. It is observed in Figure 10c that by increasing the beaconing period the clusters become larger in size and fewer in number. Number and size of clusters may also effect the energy utilization of the system, which is further investigated in Figure 11.

Figure 11a shows the remaining energy of each node at the end of simulation while using 2 s beacons. More high peaks mean more nodes are unutilized. Figure 11b shows similar results as Figure 11a but we considered have 4 s beacons.

The system is evaluated by changing the beaconing period from 1 to 4 s. If a 4 s beacon is used about 580 clusters are made in 100 rounds. Further, in the case of a1 s beacon the number of clusters is 980. Figure 11a shows that about 10 nodes are left unattended or unutilized at the end of the simulation, while in Figure 11b the energy utilization is improved in the sense that only two nodes are observed unutilized and few are underutilized.

#### 8.3.3. Dead Nodes Investigation in Matlab

**Figure 12:**
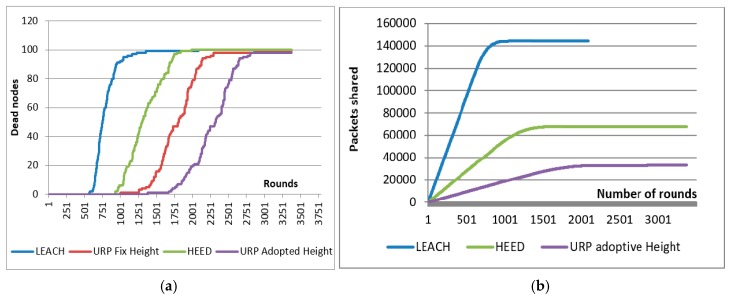
Comparison of existing routing protocols with the proposed system (**a**) Number of rounds vs. dead nodes; (**b**) comparison of the amount of inter-cluster communication in different routing algorithms that is required to formulate a cluster.

Figure 12a shows the comparison of dead nodes vs. UAV rounds in different routing schemes. The number of rounds is shown on the X-axis and number of dead nodes is shown on the Y-axis. Different colored curves represent different data gathering schemes. The lower the curve, the higher the performance. Red and purple lines show the performance of proposed UAV Routing Protocol (URP). We tested the developed system at constant and fixed height (the UAV is not going down to take data) as in case of other networks (LEACH and HEED) and the results are shown in the red curve. When the UAV is flying at a fixed constant height even then our proposed system is performing quite well. The purple curve URP-adopted height is the best case scenario when the UAV negotiates the best height with the CH in advance and respect it while collecting the data. Thanks to the fact that there are no periodic updates, no flooding of information, better CH selection and duel frequency use, that helps us to optimize the node energy up to a maximum extent. The UAV and CH negotiate a suitable height for data collection. As long-range communication is the main source of energy depletion, adjusting has in a good impact on overall system lifetime.

#### 8.3.4. Inter-Cluster Communication Assessment

Number of packets exchanged among clusters to build a network is shown in Figure 12b. The number of rounds is shown on the X-axis, packets delivered are represented by the Y-axis and the colored curves shows different protocols. The lower the curve the better the system is. It is observed that about 140 k packets are exchanged among cluster members and cluster heads in 1000 rounds just only to keep network live and updated in the form of periodic updates, clustering information and CH notifications. In the proposed system, only 20 k messages are exchanged, which is a great factor that improves the system performance a lot.

## 9. Proof of Concept

**Figure 13:**
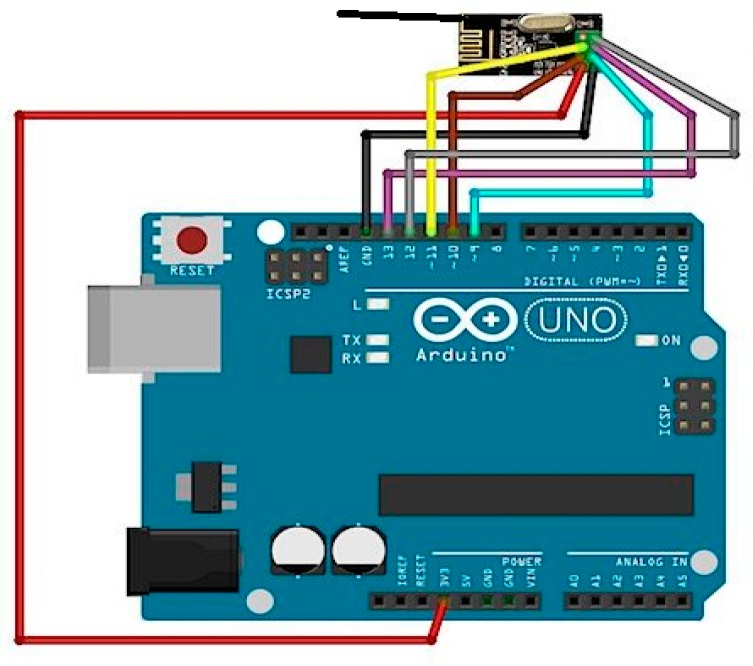
Proof of concept sensor and UAV node.

We used an Arduino microcontroller to build two components, an UAV module and ground sensor nodes. A specialized UAV is also made to carry this equipment and can operate as per instructions given onboard by our developed system. IoT and UAV sink nodes are developed using same hardware shown in Figure 13, the only difference being the software uploaded because they have different functionality.

**Figure 14:**
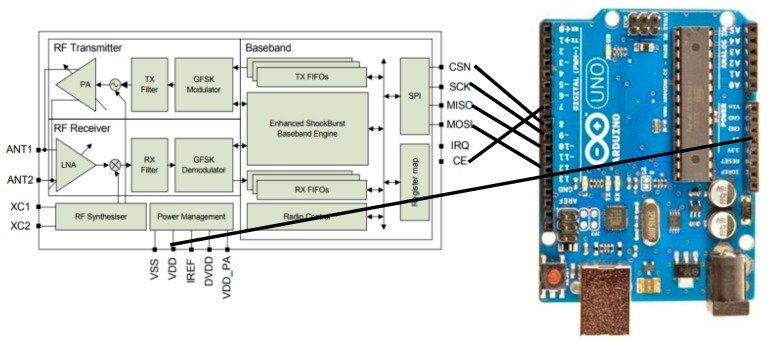
NRF24L01 and Aduino wiring diagram.

Both devices are built using a nRF24L01 transceiver, which is a low power consumption transceiver operating at 2.4 GHz frequency and capable of transmitting data at rates up to 2 Mbps. A circuit diagram for the nRF24L01 [28] and wiring information are shown in Figure 14.

Initially, we loaded all sensor nodes with 20 Kb (Kilo Bytes) of data and five nodes are deployed in the field. When the UAV approaches the field, one of them is elected as CH dynamically and it collects all the data from neighboring nodes and finally transmits 100 Kb data to the UAV.

The localization components, including the virtual antenna, UHF 433 MHz transceiver for the UAV and sensor nodes are still in development and not installed so far. The first test run results are given below. All these results do not include the UAV navigation time.

### 9.1. Working of Cluster Member Nodes

**Figure 15:**
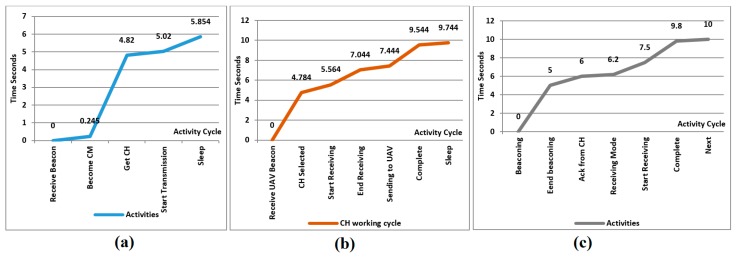
Activities of proof of concept devices related to time (**a**) sensor node (**b**) cluster head (**c**) UAV.

The algorithm described in Section 6 is developed in Arduino language which is mostly based on C++. The developed code then builds on Arduino Uno board shown in Figure 13 that is developed to make a field sensor node. We choose Arduino mini to keep the node size small. Figure 15a represents the activity graph of a ground sensor node that acts as cluster member. It is observed that it takes about 5.4 s to complete the cluster formation and data delivery. In that figure, the X-axis represents the CM sequence of activities, while the Y-axis shows the time in seconds and the curves represent the relationship betweentime and activities. The parameters of this node were set to assume it doesn’t have good specification and its CH probability is almost 0, so it decides immediately to set its status as CM. Cluster head selection takes about 4.5 s and finally data transmission takes less than a second to complete its process.

### 9.2. Cluster Head Node Activities

Figure 15b shows the graph of CH working. It takes about 10 s to complete its working cycle. It is observed that this node becomes a CH in 5 s, during this period, it becomes activated, contacts neighboring nodes and shares information with the UAV. As this node has been selected as a CH, it has to collect data from all other members and transmit the aggregated data to the UAV. This whole procedure is conducted in 5 s.

### 9.3. Working Cycle of the UAV

Life cycle of the UAV is shown in Figure 15c. It sends a beacon message in the first 5 s, then switches to discovery phase to search for a suitable CH. Once a CH is selected, it will navigate to approach it and collect data at some reasonable height. The whole procedure takes about 10 s.

### 9.4. Combined Activities Analysis

**Figure 16:**
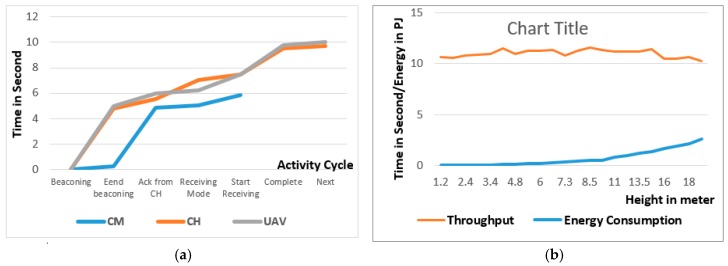
Performance analysis of proof of concept devices (**a**) activities of different components with respect of time (**b**) effect of varying height on throughput and energy consumption.

Comparison of UAV activities with respect to the CM and CH are shown in Figure 16a. The X-axis represents UAV activities, while the Y-axis shows time in seconds and different color curves represent different components (UAV, CH and CM).

### 9.5. Effect of Varying Height on Data Collection

We evaluate the effect of changing height on the system performance and results are shown in Figure 16b. The X-axis shows the height of the UAV from 1.2 m to 20 m. and the Y axis represents both time consumed in seconds and energy utilization in PJ (Peta Joules).

By increasing the height of the UAV, we could not notice any effect on the data collection time but it may have a massive impact on the CH energy utilization.

## 10. Conclusions

We have developed and tested a dynamic data collection method where data can be collected from selected nodes in a targeted area. In this project, an UAV can move freely without considering the ground network topology. To tackle this, it is proposed that ground nodes will form clusters according to the UAV’s path and the nature of data. Further we introduced a Bayesian probability- based cluster head selection process to select the best node as a cluster head. The developed system is evaluated through simulation and proof of concept devices. From the simulation results, we can conclude that the proposed system can drastically increase the network lifetime while maintaining a good throughput. For proof of concept, sensor and sink nodes are developed by using an Arduino microcontroller where the sink node is integrated with the UAV so that it can control the UAV’s flight. Real time field data is collected and different parameters are monitored for performance evaluation. The measured results showed that this system is practically feasible while offering many benefits over traditional approaches. Critical data about crops like nutritional stress, bug attacks or the spread of diseases can be detected well in time so they can be cured before the crop is affected or destroyed. By using our developed system, farmers do not need the regular visits to their large farm field and they can rely on this fully automatic system to get accurate, up-to-date and precise information. Furthermore this system can help the farmers to plan the optimum utilization of resources as the system can take appropriate action on their behalf, if some threshold is achieved. Considering all this we can say that developed system will facilitate the use of IoT technology for agriculture to take care of crop health to ensure the quality and quantity of foods. Moreover, the proposed system can be extended in the future in many ways; multiple UAVs can be used in a a coordinated way to further increase the throughput. Single hop clusters can be extended to multi-hop ones at the same time that more professional sensors and sink devices can be made from the proof of concept.

## Figures and Tables

**Table 1:** UAV beacon message.

Type (6 bytes)	Height (2 bytes)	Threshold (1 byte)	Other (2 bytes)
Header		Payload	Trailer

**Table 2:** Sensor noder unique ID.

Circle No.	Type	Sub Type	Purpose	Unique No.	Total Size
1 byte	1 byte	1 byte	1 byte	2 bytes	6 bytes
Prefix					

**Table 3:** Sensor node reply.

ID (6 bytes)	Probability (2 bytes)	Trailer (1 byte)
Header	Payload	Trailer

**Table 4:** The list of variables used in state diagram.

Variable	Meaning
CCH	Set of cluster heads
CID	Id of CH broadcast by UAV
$D_{1}$	Distance for localization at least 120 m we are taking it 200 m
$D_{2}$	Distance for data collection (95 m)
$D_{2L}$	Minimum possible distance for data collection (170 m)
$D_{2H}$	Maximum possible distance for data collection (170 m)
$E_{i}$	Energy of node *i*
$F_{1}$	1st Frequency 433 MHz
$F_{2}$	2nd Frequency 2.4 GHz
g	GPS accuracy
$ID_{i}$	Id of crop sensor *i*
${𝒷}_{i}$	Data of node *i*
${ℒ}_{i}$	Location of node *i*
m	Number of antenna elements
n	Number of Sensor nodes (S1, S2, …, Sn)
$P{ℒ}_{i}$	Probability of node *i* by known its location
q	Number of CCH nodes
T𝒷	Total data of a cluster
Th	Threshold set by UAV, default = 0
$TE_{Tx}$	Total energy requires to send T𝒷 bits to $D_{2}$ distance
$T_{ys}$	Type of sensor node announce by UAV
$P_{i}$	Probability of node *i* to become a cluster head

## References

[1] Fu C., Jiang Z., Wei W., Wei A. (2013). An Energy Balanced Algorithm of LEACH Protocol in WSN. Int. J. Comput. Sci..

[2] Kaixin X., Gerla M. A Heterogeneous Routing Protocol Based on a New Stable Clustering Scheme. Proceedings of the Military Communication Conference MILCOM.

[3] Shangguan L., Mai L., Du J., He W., Liu H. Energy-efficient Heterogeneous Data Collection in Mobile Wireless Sensor Networks. Proceedings of the 20th International Conference on Computer Communication Networks (ICCCN’2011).

[4] Khan A.W., Abdullah A.H., Razzaque M.A., Bangash J.I. (2015). VGDRA: A Virtual Grid-Based Dynamic Routes Adjustment Scheme for Mobile Sink-Based Wireless Sensor Networks. IEEE J. Sens..

[5] Okcu H., Soyturk M. (2014). Distributed Clustering Approach for UAV Integrated Wireless Sensor Networks. Int. J. Ad Hoc Ubiquitous Comput..

[6] Khan A., Abdullah A., Anisi M., Bangash J. (2014). A Comprehensive Study of Data Collection Schemes Using Mobile Sinks in Wireless Sensor Networks. IEEE J. Sens..

[7] Chang J.-Y., Ju P.-H. (2012). An Efficient Cluster-based Power Saving Scheme for Wireless Sensor Networks. EURASIP J. Wirel. Commun. Netw..

[8] Sharef B.T., Alsaqour R.A., Ismail M. (2014). Vehicular Communication Ad-hoc Routing Protocols: A Survey. J. Netw. Comput. Appl..

[9] Duan Y.E. Design of Intelligent Agriculture Management Information System Based on IoT. Proceedings of the 2011 Fourth International Conference on Intelligent Computation Technology and Automation.

[10] Fan T.K. (2013). Smart Agriculture Based on Cloud Computing and IOT. J. Converg. Inf. Technol..

[11] Bo Y., Wang H. The Application of Cloud Computing and the Internet of Things in Agriculture and Forestry. Proceedings of the 2011 International Joint Conference on Service Sciences.

[12] Uddin M.A., le Jeune D., Mansour A., el Aggoune H.M. Direction of Arrival of Narrowband Signals Based on Virtual Phased Antennas. Proceedings of the IEEE 23rd Asia-Pacific Conference on Communication.

[13] Koutsias J., Chandrinos K.V., Paliouras G., Spyropoulos C.D. An evaluation of Naive Bayesian anti-spam filtering. Proceedings of the 11th European Conference on Machine Learning.

[14] Mokhesi L., Bagula A. Context-aware handoff decision for wireless access networks using Bayesian networks. Proceedings of the Annual Research Conference of the South African Institute of Computer Scientists and Information Technologists.

[15] Domingos P., Pazzani M. (1997). On the Optimality of the Simple Bayesian Classifier under Zero-One Loss. Mach. Learn..

[16] Wang Q., Garrity G.M., Tiedje J.M., Cole J.R. (2007). Naive Bayesian Classifier for Rapid Assignment of rRNA Sequences into the New Bacterial Taxonomy. Appl. Environ. Microbiol..

[17] Androutsopoulos I., Koutsias J., Chandrinos K.V., Spyropoulos C.D. An experimental comparison of naive Bayesian and keyword-based anti-spam filtering with personal e-mail messages. Proceedings of the 23rd Annual International ACM SIGIR Conference on Research and Development in Information Retrieval.

[18] Faruque S. (2015). Chapter 2 Free Space Propagation. Radio Frequency Propagation Made Easy.

[19] 2.4 GHz IEEE 802.15.4/ZigBee-Ready RF Transceiver. http://www.ti.com/lit/ds/symlink/cc2420.pdf.

[20] CC1021 Single Chip Low Power RF Transceiver for Narrowband Systems. http://www.ti.com/lit/ds/swrs045e/swrs045e.pdf.

[21] Ammad-udDin M. (2017). Field Test of Agriculture IoT Devices. https://www.youtube.com/watch?v=Bma2qE7f1gk.

[22] Ammad-udDin M. (2017). UAV Connectivity with Sensor Nodes. https://www.youtube.com/watch?v=xzf4SkhDteE.

[23] Ammad-udDin M. (2017). STK Simulation of Dynamic Data Collection by UAV. https://www.youtube.com/watch?v=OXtNn94l1xA.

[24] Ammad-udDin M. (2017). MatLab 3D Simulation of Dynamic Clustering. https://www.youtube.com/watch?v=zVM42i9BQFY.

[25] Ammad-udDin M. (2017). OMNet ++ Simulation of Dynamic Clustering and Crop Health Monitoring. https://www.youtube.com/watch?v=pYx-Z0chWoA.

[26] Li X., Nayak A., Stojmenovic I. Exploiting Actuator Mobility for Energy-Efficient Data Collection in Delay-Tolerant Wireless Sensor Networks. Proceedings of the 2009 Fifth International Conference on Networking and Services.

[27] Rao J., Biswas S. Joint Routing and Navigation Protocols for Data Harvesting in Sensor Networks. Proceedings of the 5th IEEE International Conference on Mobile Ad Hoc and Sensor Systems.

[28] RF24L01 2.4GHz Radio/Wireless Transceivers How-To. https://arduino-info.wikispaces.com/Nrf24L01-2.4GHz-HowTo.

